# On the Degeneracy of the Teeth

**Published:** 1865-07

**Authors:** H. H. Winn


### ON THE DEGENERACY OF THE TEETH.

BY H. H. WINN.

To the question, is there a degeneracy of the teeth now as compared with former years? I think there can be but an affirmative answer.

It is true, that in the absence of dental data and dental records that afford us any *direct* and *positive* light on this subject, we should be somewhat at a loss to derive from these alone, any conclusive and authoritative evidence, that there was indeed a marked degeneracy of the teeth of the present, as compared with former generations.

Yet, should *twenty-five* years be taken as the initial of our review, we will not have passed beyond the period of persons now living, who were then on the stage of life and occupying a similar position with their children, now in the middle of life, whose mouths we might compare together in substantiation of our proposition, that *there is a degeneracy of the teeth.*

True, in so short a period as twenty-five years, there can and will be in many cases no real marked contrast, and much of those same general features will be observed, which would be attributable to hereditary influence—that is, the children will represent their parents; and consequently this generation would represent the preceding.

And it is for this reason, that we have instituted this comparison, to see how closely such a comparison holds good.

Of course, in a practice so limited as my own, I have not been able to trace this degeneracy of the teeth very far back.

But I have had several marked cases, when children's teeth were presented for examination, and their parents would immediately ask the question—"Why is it that I have but few decayed teeth comparatively, at the age of thirty, whereas, my child has a number of decayed teeth, not having reached *half my age.*"

In the most of these cases, I have noticed that hereditary tendency has generally been observed; and the peculiarities that sometimes over-leap one generation, to find in the cross of the third the conditions of temperament favorable to their development—and some times, when peculiarities would descend continuously in the same family, especially where the family lineaments were preserved in a marked degree.

But we do not desire to do more than to admit the affirmative reply to this part of our subject; and therefore shall pass it over without further investigation.

The extreme frailty of the human teeth of the present age, especially in the rising generation, very naturally directs the inquiring mind to the investigation of the *causes* of this greatly increased degeneracy. Indeed, as I remarked before. I have been often asked to define the difference in the structure of the children's teeth and their perfect development, that *now* come under the care of the Dentist; which so strongly characterise them from their fore-fathers, and I have often been at a loss to give any satisfactory answer to the question. Accordingly, I have tried to investigate this subject to only a limited extent, and hoping for all leniency, shall endeavor therefore to elucidate my subject as clearly as I can. Man in his original state, or when he is not influenced in his physical well-being by the customs—or more really, by the requirements of highly cultivated and civilized life—not as directed by the unerring light of science—but as his perverted taste, and the artificial customs of this age would incline him—had a superior physical organization; and accordingly he was better able to withstand the exciting causes of disease.

He lived plainly upon the various articles of food which *nature* seemed to provide for his best needs. And I firmly believe, that as man's rules of life are influenced by injurious and unnatural tastes and desires, to such a proportion will he be physically degenerated and inclined or predisposed to disease.

Of course, the strong and vigorous constitution, has within itself the means of defense against the inroads of disease; but the weakly on the other hand, having no such power of resistance, is brought to suffer all the maladies to which it is hereditarily or otherwise predisposed. I wish therefore now to proceed to apply myself to the special consideration of the *causes* of this degeneracy.

1st. This degeneracy I think results from a general interruption of the functions of nutrition during the formation of the teeth.

My remarks on these causes of degeneracy, I wish to be applied to the *mother*, who is appropriating from her blood elements of tissues for the construction of a new being, as long as she is physically related to it—during *Gestation* and *Lactation.*

Indeed, I consider this as one of the most interesting and important phemomena of nature, as regards causes and effects, that is prsented for the study and investigation of the earnest mind—and though I have made but little progress myself, yet I feel that there is room for very extended observation; and that such labors can not go unrewarded.

At birth, we find contained within the alveolar ridges of the upper and lower Maxillary bones the germs of *fifty-two teeth.* The foundation for all these teeth have been laid during *Gestation*—before the child is born. We know too, that the "fœtus" derives its life and growth from the blood of its mother, and consequently we must look to the *mother* to supply that blood with the necessary ingredients, which shall give healthy and necessary development to these teeth which shall perform so important functions in the economy of nature.

Now can not we trace the degeneracy of the teeth to the *imperfect food* which goes to make up the ingredients of the blood, which gives growth and development to these teeth.

It is said, "that a minute examination of the teeth presents this truth; that in proportion to the relative deficiency of *earthy* constituents, are they acted upon by the ordinary exciting causes of caries—and that the deficiency of the *Phosphate of Lime* measures their predisposition to that disease."

Now, is there not a deficiency of Phosphate of Lime.

1st. *In the preperation of some kinds of the ordinary food in common use?* One writer asserts, that the early decay of the human teeth and the rapidity of dental caries is attributable to the close bolting of flour in its manufacture—and I am inclined to believe from inquiring, that not only flour but most of the other brands of the cereals are manufactured from the *white* varieties of grain, which contain less *gluten* than the yellow varieties; and are therefore less productive of strength to the organization. I have not however been able to carry this investigation to such a point, as to be able to discover *just what* preperations of food are most productive of Lime.

But that the system obtains its Phosphotes of Lime from the food, is certain; and it is quite certain also, that it would be impossible for the animal economy to carry on with perfectness and strength the process of nutrition, unless the food were properly prepared, and this leads me to speak secondly of the *quality of the food.*

The quality of the food is of course closely allied with its preperation—because the quality of the food, for the proper and healthy nutrition of the bones and teeth depends upon the quantity of the Phosphates of Lime also,—that is—for the *usual* supply of this Lime I mean.

But this quantity of Lime varies so much in different articles of food, that I am inclined to consider this also as an important cause of the degeneracy of the teeth.

We know the small quantity of Phosphates which beans, peas and most of the Lentils contain, and that they are consequently of minor importance as articles of nourishment—hence, they satisfy the appetite only without increasing the strength.

On the contrary, "corn, milk and the flesh of animals" is said to furnish the Phosphates to a greater extent. I can not enter into a more extended investigation of this subject, as to the exact proportion of the different kinds of food in common use should be used, in order that the mother's blood may be furnished with the right kind of constituents for the proper development of the teeth structure.

Suffice it to say, that I think this part of a mother's duty is often seriously neglected—and it is indeed an important matter that her blood should be furnished with a greater quantity of the Phosphates and other earthy elements than her own system requires—because not only during *Gestation*, but also during *Lactation*, the *milk* must possess this same ingredient, for the healthy organization and development of the teeth.

I believe the selection and preperation of the food has more influence over the human organism and physical health than is generally supposed.

Would it not then be the highest attainment to be able to dictate to the pregnant and nursing mother those hygienic rules, which will enable her to give to her infant that intrauterine and early development which will be likely to insure it health in after life; and not bring it forth already predisposed to disease.

The second main cause of this degeneracy of the teeth, is owing to the difference of the general modes of living now, as compared with former times.

Caries did not attack the teeth of persons of forty and fifty years ago, as early as it does now.

The delicate young girl of "affluent" city parents cuts her first permanent molar at six, and often has them extracted already *mere stumps* at eight and ten years of age; and indeed it is a rare thing for any person. male, and especially female, to reach the age of eighteen or twenty without losing more or less of their natural teeth, unless they have been prevented from further decay by "filling."

The inhabitants of this country, say for the first one hundred years, lived under nearly the same influences as to exercise in the open air, diet, &c., as they had been exposed to before their emigration.

The *coarse* cereals were then used—they were healthly from vigorous and out-door exercise, and a coarse but healthy diet. Abstinence from highly spiced food and condiments, together with an abundance of wholesome exercise, seem to constitute the main difference between them and the young of to-day.

Had climate any thing to do with it, it surely would long since have affected the Indians. But on the contrary, they possess very good teeth comparatively, which show no signs of decay unless injured by habit of civilization.

The same characteristics are remarked in the teeth of the Negro—most of them seem to have perfect teeth as far as I have been able to judge; and I think that the same reasons can be given for the uniform good condition of their teeth, as for the teeth of the Indian.

Consequently, we infer that the degeneracy of the teeth, as well as their inferiority at the present day, must be attributable to inherent weakness, or to acquired vices and the many unnatural customs which are so apparent in our day.

